# Patient reported toxicity and quality of life after hypofractionated high-dose intensity-modulated radiotherapy for intermediate- and high risk prostate cancer

**DOI:** 10.1016/j.ctro.2021.05.005

**Published:** 2021-05-21

**Authors:** Jeroen Houben, Gill McColl, Johannes HAM Kaanders, Robert J. Smeenk

**Affiliations:** Department of Radiation Oncology, Radboud University Medical Center, P.O. Box 9101, 6500 HB Nijmegen, the Netherlands

**Keywords:** Prostate cancer, Radiotherapy, Hypofractionation, Patient reported outcomes, Health related quality of life

## Abstract

**Background and purpose:**

For irradiation of localized prostate-cancer, moderately-hypofractionated regimens with a variety of dose per fraction are used. We adopted a regimen of 70 Gy in 28 fractions of 2.5 Gy, using state of the art radiotherapy (RT) and closely monitored the efficacy, toxicity and health-related quality of life (HRQoL) in a large cohort, using patient-reported outcomes.

**Materials and methods:**

Between 2008 and 2016, 462 patients with intermediate- to high-risk localized prostate cancer were treated with RT, 28 fractions of 2.5 Gy, using IMRT/VMAT, an online fiducial-maker based correction protocol and a daily inserted endorectal balloon. Overall freedom from failure (no biochemical or clinical recurrence) , as well as self-reported genitourinary (GU) and gastrointestinal (GI) related toxicity and HRQoL are reported.

**Results:**

Overall freedom from failure rates at 3 and 5 years were 92.0% (89.1–94.9%) and 83.5% (78.6–88.4%), respectively. Prevalence rates of grade ≥ 2 GU/GI-toxicity were 16.3%/6.3% and 22,1%/3.2% after 3 and 5 years respectively. The 5-year actuarial incidences of grade ≥ 2 GU/GI-toxicity were 43.5%/18.5%. HRQoL worsened during RT and gradually recovered thereafter, In accordance with the prevalence rates.

**Conclusion:**

Treatment of intermediate- or high-risk localized prostate cancer with RT to 70 Gy in 28 fractions with IMRT/VMAT, using fiducial markers and an endorectal balloon leads to good long-term tumor control rates and acceptable patient reported toxicity rates. Furthermore, patient-reported outcomes, including HRQoL, are essential for a good comparison between different studies. Finally, prevalence rates show a better correlation with HRQoL than actuarial incidence rates do and might therefore better represent the burden of toxicity.

## 1 Introduction

Dose escalation leads to an improved overall survival in patients with intermediate- and high-risk prostate cancer (PCa) [1], [2], [3]. As a consequence, late toxicity and health related quality of life (HRQoL) after treatment have become increasingly important.

In order to reduce toxicity while escalating the tumor dose, several methods have been developed to minimize the radiation dose to surrounding organs at risk (OARs). Intensity-modulated radiotherapy (IMRT), including volumetric arc therapy (VMAT) [4], [5], the daily use of fiducial markers implanted in the prostate[6], [7] and the use of daily inserted endorectal balloons (ERB) during radiotherapy (RT) [8], [9] all contribute to a lower dose at the OARs, especially the rectum.

Clinical-radiobiologic studies have shown that PCa is likely to have an α/β-ratio below 2.0 Gy [10], [11], [12], which is lower than its surrounding tissues. This has led to the introduction of hypofractionated RT, thereby escalating the biological equivalent dose (BED) to the tumor, without increasing the BED to the surrounding tissues. Multiple studies have shown non-inferiority of moderately hypofractionated regimens in terms of efficacy with acceptable toxicity profiles [13], [14], [15]. While in all risk groups moderately hypofractionated RT is advocated, in low- and intermediate risk PCa even extremely hypofractionated (≥5 Gy per fraction) treatments can be considered based on recent publications [16].

In 2005 Kupelian et al. reported their long-term outcomes, using a total dose of 70 Gy in 28 fractions of 2.5 Gy in low-, intermediate- and high-risk localized PCa. This study showed excellent results for both biochemical failure free survival and late toxicity [17]. This has resulted in the adaptation of this schedule as the standard treatment for PCa in our institution, while closely monitoring toxicity and health-related quality of life (HRQoL)

Most reported toxicity rates are based on physician-reported outcomes. However, these can underestimate both the frequency and the severity of toxicity compared with patient-reported outcome measures (PROMs) [18]. Moreover, PROMs better reflect the true burden for the patients and their HRQoL.

In this paper PROMs and HRQoL scores, both during and after RT, are reported in a large cohort of consecutive PCa patients treated with IMRT/VMAT-based moderately hypofractionated high-dose RT who were prospectively followed using validated questionnaires. In all patients state-of-the-art fiducial marker-based correction protocols and a daily inserted ERB were used.

## 2 Methods

### 2.1 Patient selection

Between June 2008 and February 2016 all consecutive patients with EAU-ESTRO-SIOG medium- or high-risk localized PCa who were treated with IMRT radiotherapy were handed out self-questionnaires at baseline, during treatment and during follow-up [19]. This was approved by the local medical ethics committee.

### 2.2 Treatment procedures

All patients were treated, according to local protocol, with external beam RT to a prescribed dose of 70 Gy in 28 fractions (biologically equivalent to 78–80 Gy in fractions of 2 Gy with an estimated α/β of 1.5–2.0 Gy) [17], 4 fractions per week with an overall treatment time of 46–49 days. Treatment was in supine position with IMRT (including VMAT) using online fiducial-marker based electronic portal imaging. Patients received upfront bladder instructions for both the planning-CT-scan and all treatment fractions. Also, an air-filled (100 cc) ERB (QLRAD B.V. The Netherlands), was introduced into the rectum prior to the CT scan and all treatment fractions.

Three treatment groups were defined based on the risk of seminal vesicle involvement according to the Partin tables [20]. In group 1 (<10% risk), the CTV consisted of only the prostate. In group 2 (10–25% risk) it also included the base of the seminal vesicles. In group 3 (>25% risk), it included the entire seminal vesicles. Elective lymph node areas were not included in the CTV. The PTV included the CTV with a 5 mm posterior margin and a 7 mm margin in all other directions.

The prescribed dose to the PTV was 70 Gy in 28 fractions, requiring > 99% of the PTV to receive at least 95% of the prescribed dose. Dose constraints for the combined anal and rectal wall were a maximum of the volume receiving ≥ 30 Gy , ≥40 Gy and ≥ 60 Gy of 80%, 60% and 30% respectively and a mean dose of ≤ 45 Gy to the rectal wall and ≤ 30 Gy to the anal wall. For the urinary bladder a maximum volume of 50% receiving ≥ 60 Gy was permitted and ≤ 10% of the individual femoral heads could receive ≥ 50 Gy.

### 2.3 Questionnaires

The questionnaires consisted of the Expanded Prostate cancer Index Composite (EPIC) [21] for HRQoL combined with complementary questions concerning the genitourinary (GU) and gastrointestinal (GI) toxicity [22] (complementary questions can be found in the supplementary material). Patients were asked to fill out these questionnaires 6 weeks, 6 months and every year up to 5 years after RT. As of march 2011 all patients were also asked to fill out questionnaires at baseline and during week 5 of radiotherapy. Questionnaires were sent to the patients’ home address and were either handed back during follow-up visits or sent back through mail. Follow up was performed alternating between the radiation oncologist and the referring urologist, or only by the referring urologist at 6 weeks, then every 3 months for the first year followed by once every 6 months until 5 years after RT. Every visit included a medical history and a serum prostate specific antigen (PSA) assessment.

### 2.4 Scoring of endpoints

For toxicity scoring a previously described method was used [22], [23], in which the questionnaires (including both PROMs and additional questions) were used to classify the symptoms for multiple subdomains of GU and GI toxicity according to the Radiation Therapy Oncology Group/European Organization for Research and Treatment of Cancer (RTOG/EORTC) scoring system. The GU and GI toxicity reported represent the highest grade scored in any of their respective subdomains. For GU toxicity these subdomains are frequency, nocturia, hematuria, dysuria, obstruction, incontinence and ulceration and for GI toxicity these were frequency, blood loss, pain, obstruction, incontinence and ulceration.

For the HRQoL scoring we used the standard EPIC score calculation [21], in which each item was transformed linearly to a scale ranging from 0 to 100, with 100 being the best possible score. Domains and subdomains were then formed based on the average score of their underlying items. We only used the domains urinary summary and bowel summary and their subdomains: urinary function, urinary bother, urinary incontinence, urinary irritative/obstructive, bowel function and bowel bother. The sexual and hormonal domains fall beyond the scope of this paper.

For the prevalence analyses, we grouped both the toxicity scoring and the EPIC scores as acute phase (1–3 months after RT), 6 months (3–9 months), 1 year (10–17 months), 2 years (18–29 months), 3 years (30–41 months), 4 years (42–53 months) and 5 years (54–66 months) after RT.

Patients who did not fill out at least two questionnaires with a time-interval of at least 3 months were excluded from analysis.

Treatment failure was defined as biochemical or clinical failure, whichever occurred first. A biochemical failure was defined as an increase in serum PSA of>2.0 ng/mL above the PSA-nadir (confirmed after at least a two-week interval) [24]. Clinical failure was defined as pathological evidence of locoregional or distant recurrence of PCa or agreement of recurrence on a multidisciplinary tumor board.

### 2.5 Statistical analyses

The IBM SPSS Statistics for Windows, version 22.0 (IBM. Corp Released 2013) software was used for statistical calculations.

The freedom from failure (FFF) rate and the incidence of grade ≥ 2 GU and GI toxicity were analyzed by the Kaplan-Meier method. Follow-up for FFF ran from the end of RT to biochemical or clinical failure. Patients were censored when they were lost to follow-up or died of any cause other than PCa. The prevalence of toxicity was analyzed using descriptive statistics. For the analysis of the HRQoL scores and the change from baseline we used the Paired-Samples T-Test.

The following variables were analyzed for their univariate and multivariate prognostic value for grade ≥ 2 toxicity in a Cox’s proportional hazards model (α = 0.05): age (<70 vs. ≥ 70 years), Gleason Score (<7 vs. ≥ 7), risk-group (intermediate vs. high-risk), treatment group (including vs. excluding the (base of) the seminal vesicles), pre-treatment PSA (<20 vs. ≥ 20 ng/mL), use of (neo-) adjuvant hormonal treatment (1–6 months vs. > 6 months), use of oral anticoagulants, prior pelvic surgery and symptoms of grade 1 or worse toxicity at baseline. A non-parametric test (Spearman test) was used for comparing whether grade ≥ 2 GU toxicity was correlated to grade ≥ 2 GI toxicity.

## 3 Results

Of the 477 who started treatment in this period, 462 patients were handed out the questionnaires. 65 patients were excluded for either not filling out two questionnaires or having nodal or distant metastasis before the start of treatment, leaving 397 patients for evaluation. Their baseline characteristics are shown in Table 1.

**Table 1:** Baseline characteristics.

characteristic	n	(%)
Age		
<60	22	(5.5)
60–70	164	(41.4)
>70	211	(53.1)
PSA		
≤ 10 ng/mL	198	(50.1)
> 10 ng/mL	199	(49.9)
Gleason Score		
≤ 7	293	(73.8)
≥ 8	104	(26.2)
Risk-group		
Intermediate	134	(33.8)
High	263	(66.2)
Clinical T-stage		
≤2	182	(45.8)
≥3	215	(54.2)
Hormonal treatment		
≤ 6 months	145	(36.5)
> 6 months	48	(12.1)
Diagnostic PLND	120	(25.7)
Treatment group		
prostate only	63	(15.9)
prostate + base of SV	222	(55.9)
prostate + whole SV	112	(28.2)
Use anticoagulants	47	(11.9)
Use of antiaggregants	104	(26.2)
Comorbidity		
diabetes	44	(11.1)
hemorrhoids	31	(7.8)
intestinal polyps	10	(2.5)
major abdominal surgery	34	(8.8)

Abbreviations: PSA (prostate specific antigen), PLND (pelvic lymph node dissection), SV (seminal vesicles)

Median follow up was 45 months (range: 6–103 months). The FFF rate at 3 and 5 years were 92.0% [95% CI 89.1–94.9%] and 83.5% [78.6–88.4%] respectively. For the intermediate- and high-risk tumor groups these rates were: 94.9% [90.7–99.0%] vs. 92.3% [88.8–95.8%] at 3 years (p = 0.22) and 89.1% [81.7–96,5%] vs. 81.0% [74.9–87.1%] at 5 years (p = 0.12).

**Fig. 1:**
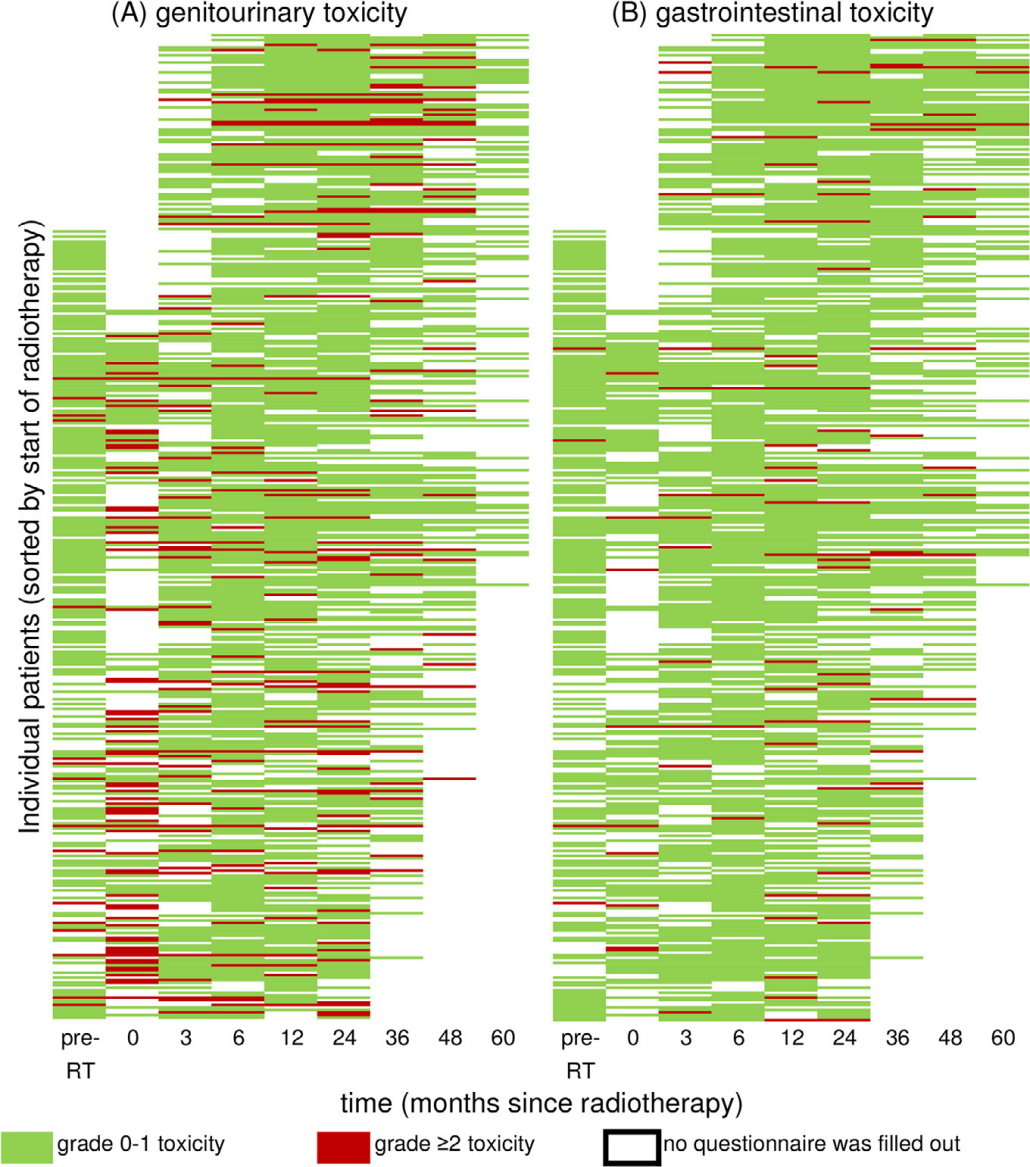
Grade 0–1 vs. grade ≥ 2 **(A)** genitourinary toxicity and **(B)** gastrointestinal toxicity for individual patients at each individual measuring moment, scored with self-questionnaires. a*bbreviations:* pre-RT = pre-radiotherapy.

The response rates for the questionnaires were 57.7% at baseline (including patients that did not receive a baseline questionnaire), 38.8% during RT (including patients that did not receive a baseline questionnaire), 61.1% in the acute phase, 82.1% at 6 months, 79.6% at 1 year, 78.8% at 2 years, 56.9% at 3 years, 39.5% at 4 years and 24.4% at 5 years. Patients filled out a median of 5 questionnaires (range 2–9). Fig. 1 shows the respective questionnaires filled out by the individual patients as well as whether they scored grade ≥ 2 GU or GI toxicity at the different follow-up intervals.

**Fig. 2:**
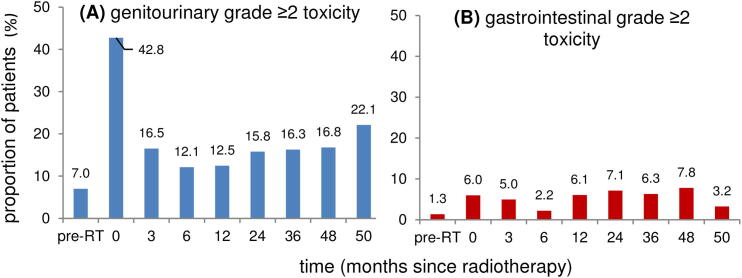
Prevalence over time of grade ≥ 2 **(A)** genitourinary toxicity and **(B)** gastrointestinal toxicity scored with self-questionnaires before and during radiotherapy and at different follow-up intervals.

The prevalence rates of grade ≥ 2 GU and GI toxicity are shown in Fig. 2. The prevalence of grade ≥ 2 GU toxicity was 7.0% at baseline, peaked during RT, decreased again at 6 months and thereafter slowly increased to 22.1% at 5 years. Overall, GI toxicity was lower than GU toxicity and changes were less outspoken. The prevalence of the different subscales is shown in the supplementary material.

**Fig. 3:**
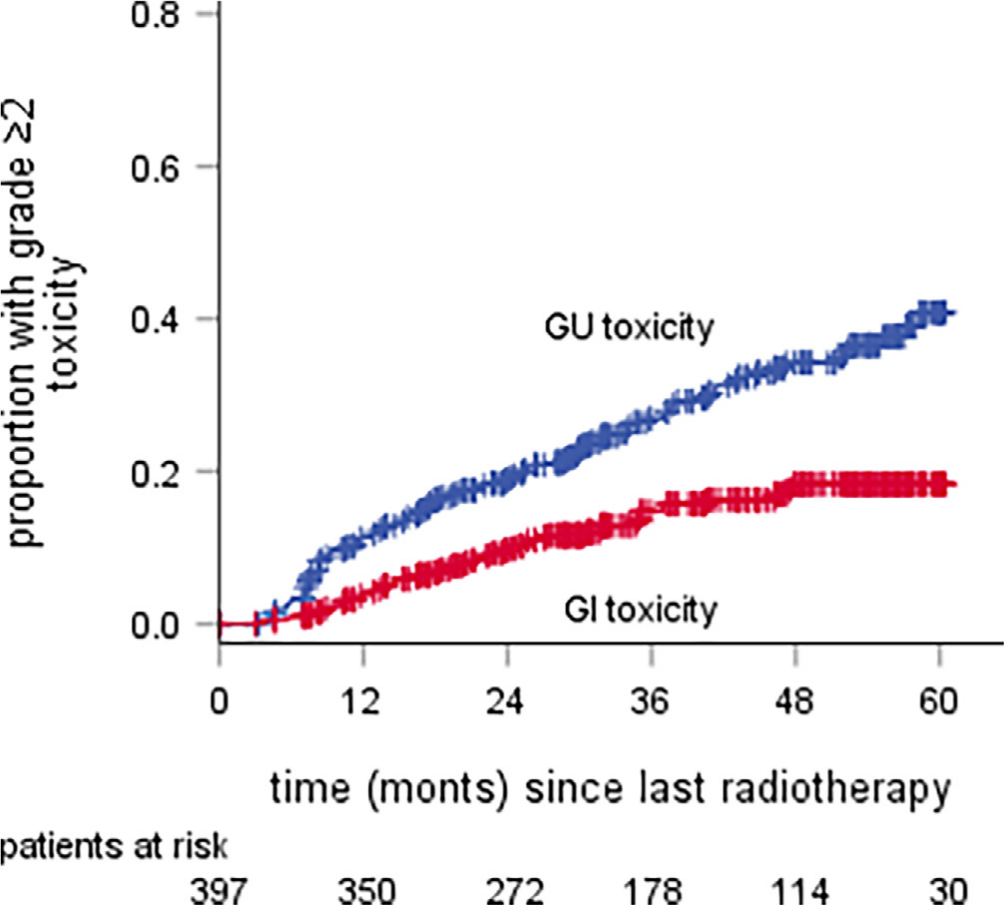
actuarial incidence rate of grade ≥ 2 genitourinary and gastrointestinal toxicity scored with self-questionnaires. *Abbreviations: GU = genitourinary, GI = gastrointestinal.*

The cumulative incidences of both GU and GI late grade ≥ 2 toxicity are shown in Fig. 3. For GU toxicity this was 43.5% [95% CI 35.3–51.7%] at 60 months and for GI toxicity 18.5% [13.8–23.2%].

**Fig. 4:**
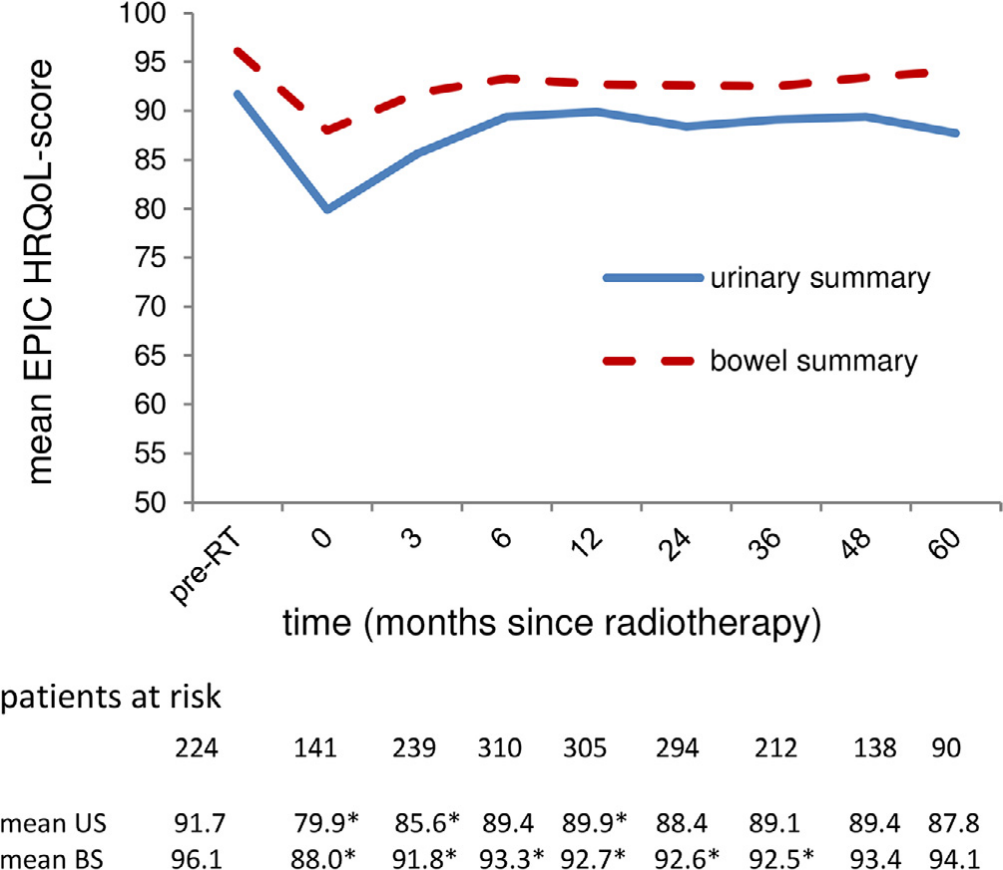
Mean health-related quality of life score, measured with the Expanded Prostate Cancer Index Composite (EPIC) for the domains urinary summary and bowel summary. Abbreviations: pre-RT = pre-radiotherapy; US = urinary summary; BS = bowel summary; * there was a statistically significant difference compared to the baseline score.

Fig. 4 shows the mean HRQoL scores for the different domains. The scores for all subdomains are shown in the supplementary material. Patients reported a baseline mean score of 91.7 [95% CI 90.4–92.93%] for the urinary domain and 96.1 [95.3–97.0%] for the bowel domain. For both domains and all of their subdomains the scores declined (p < 0.01) during RT, after which they recovered until 6 months after RT and then stabilized, although remaining slightly below the values at baseline (p < 0.01). For the urinary domain and subdomains the scores at 60 months had all somewhat declined compared to the scores at 6 months, although this was only statistically significant for the urinary incontinence subdomain (p = 0.01). This is the only subdomain where the scores at 24 and 60 months were even lower than during treatment (p < 0.01)

On univariate analysis, the following variables were statistically significant for predicting GU toxicity: treatment group (p = 0.017), prior pelvic surgery (p = 0.001) and baseline symptoms of grade 1 or worse GU toxicity (p < 0.001). On multivariate analyses prior pelvic surgery (p = 0.014) and baseline symptoms of grade ≥ 1 GU toxicity (p < 0.001) remained statistically significant. Treatment group was borderline significant (p = 0.51) For GI toxicity the significant variables on univariate analyses were: a Gleason score ≥ 7 (p = 0.050) and baseline symptoms of grade ≥ 1 GI toxicity (p = 0.021). On multivariate analysis a Gleason score ≥ 7 (p = 0.022) and baseline symptoms of grade ≥ 1 GI toxicity (p = 0.009) remained statistically significant for predicting GI toxicity. Finally, there was a significant correlation between the development of grade ≥ 2 GU toxicity and grade ≥ 2 GI toxicity (p = 0.008).

Given the fact that not all patients filled out all the questionnaires during follow-up, we did exploratory analyses in a subcohort of patients who filled out ≥ 6 out of 7 questionnaires at baseline and 6, 12, 24, 36, 48 and 60 months after RT to see whether the abovementioned toxicity rates and HRQoL scores give a fair representation. This group consisted of 96 patients. The results from these analyses are shown in the Supplementary material. Overall, the outcomes were very similar to those of the total cohort. The FFF rates were 96.8% [95% CI 93.6–100.0%] and 88.2% [85.0–91.4%] at 3 and 5 years respectively. The prevalence rates of grade ≥ 2 toxicity were 14.7% and 23.9% for GU toxicity and 7.5% and 3.1% for GI toxicity at respectively 3 and 5 years after RT. The cumulative incidence of grade ≥ 2 toxicity at 5 years after RT was 40.2% [95% CI 51.2–29.2%] for GU toxicity and 15.6% [22.2–9.0%] for GI toxicity. Finally the EPIC HRQoL scores were 88.8 [95% CI 86.5–91.0] and 86.9 [84.1–89.9] for the urinary summary domain and 92.9 [91.0–94.8] and 94.2 [91.7–96.7] for the bowel summary domain at 3 and 5 years respectively.

## 4 Discussion

This study shows that a moderately hypofractionated regimen of 28 times 2.5 Gy using state-of-the-art RT with fiducial markers and an ERB leads to good tumor control rates and very acceptable patient-reported toxicity rates in patients with intermediate and high-risk PCa. The FFF-rates are in line with those of other studies using moderately hypofractionated RT [14], [15], [17].

The literature presents a wide range of actuarial cumulative incidence rates of grade ≥ 2 GU/GI toxicity after moderately hypofractionated RT. The rates in this paper, 26%/15% after 3 years, compare favorable to those of the Dutch HYPRO-trial: 41%/22%, and the rates of 44%/19% after 5 years are in line with those of Pollack et al. [25]: 39%/18%. On the other hand, several authors report lower toxicity rates, like Kupelian et al.: 11%/11%, 5 years after treatment [17]. However, in most of these papers only physician-reported toxicity was reported, which tends to result in lower toxicity rates when compared to patient-reported outcomes [18] .

Noteworthy, Dearnaley et al., using physician-reported outcomes, present rates ≤ 15% for both GU and GI toxicity for their trial-arms of 19–20 fractions of 3.0 Gy [14]. These are clearly lower than the rates published in this paper. However, their observed perceived urinary- and bowel complaints (16%/15%), for which they used the same self-questionnaire as in our study [14], are very similar to the ones in this paper (19%/9%). Such a difference in physician-reported late toxicity between both studies without a worse perceived urinary bother score and an even more favorable bowel bother score in the current study is an inconsistency that can only be explained by variations in interpretation and rating of toxicity among physicians. When we compare our HRQoL to other studies using the EPIC self-questionnaire, we find very comparable results [26], [27]. This points out that comparing toxicity rates between different studies is difficult, particularly when different scoring instruments were used.

Not only differences between patient- and physician-reported outcomes measuring will influence the outcomes, also the type of self-questionnaire and the translation of these questionnaire into toxicity grades will influence the toxicity rates being reported. In this study we used a widely used questionnaire for scoring the HRQoL which has a standardized method for scoring. This resulted in outcomes comparable to other papers. For the translation into toxicity, however, we used previously published methods which are much less frequently used [22] and may therefore be less suitable to use for comparison. One example is the way in which the subscale urinary incontinence was scored. Patients scored the number of days per week for which they used incontinence pads, which was translated into grades of toxicity, with a grade 3 for using pads every day. Some patients who reported to use pads every day reported only unintentional urinary loss once or twice a week. However, because they could not predict when this would happen, they used pads every day as a precaution and therefore scored grade 3 on GU toxicity. This is probably an overestimation of the true physical toxicity and a limitation of the scoring system. Since the overall GU toxicity was based on its highest score in any of the subscales, this overestimation of urinary incontinence may lead to an overestimation of overall GU toxicity.

Beside the incidence rates, the prevalence rates of toxicity were also reported. The 5-year prevalence rates of grade ≥ 2 GU/GI toxicity were 22%/3%, compared to actuarial incidence rates of 44%/19%. This was also noted by Schmid et al., reporting 5-year prevalence rates of 5%/5% respectively and actuarial incidence rates of 19%/23% [28]. This illustrates that late toxicity can be transient. This is also illustrated in Fig. 1, where some patients have reported toxicity just once or twice, with no complaints before and after. The prevalence rates in this study also have a better correlation with the HRQoL scores compared to the actuarial incidence rates. So presenting only the cumulative incidence rates might lead to misinterpretation and overestimation of the burden of toxicity [28].

Another noteworthy inconsistency in the literature is the ratio between the rates of grade ≥ 2 GU and grade ≥ 2 GI toxicity. In this study the 5-year actuarial incidence of GU toxicity is twice as high as that of GI toxicity, 44% versus 19%. The prevalence of toxicity and the HRQOL score is also worse for GU, compared to GI at every follow-up interval. Comparable differences are also reported in some papers [25], [26], [27], [28], [29] but others report almost no difference [14], [15], [16], [17], [18], [19], [20], [21], [22], [23], [24], [25], [26], [27], [28], [29], [30]. In our study it might in part be explained by the use of the ERB. This has proven to reduce GI toxicity, but is not expected to reduce the GU toxicity [9]. An ERB was not used in most other papers.

Another difference between GU and GI toxicity is the evolution over time. The actuarial incidence of GU toxicity continues to incline up to 5 years after RT, while that of GI toxicity reaches a plateau at 3 years of follow-up. This is reflected by the prevalence (Fig. 2), showing GU toxicity to stay quite stable and to incline somewhat between 4 and 5 years of follow-up, while the prevalence of GI toxicity reaches a plateau sooner and seems to even decrease a little between 4 and 5 years of follow-up. These patterns are in line with other studies [28].

A strength of this study is that the data is prospectively collected and patient-reported and thus without bias from physicians. Therein also lies a limitation. Because toxicity was not directly scored, but derived from PROMS, comparisons to other studies are difficult te make. And, although we used a method previously used by others, only by using an internationally standardized and widely used way of scoring reliable comparisons can be made.

Another limitation to this study is the low response-rate at several follow-up intervals. This asks for caution when interpreting the data. The low response rate is in part caused by a lack of questionnaires at baseline and during radiotherapy in the first part of the cohort and a lack of 4 and 5-year follow up in the last part of the cohort. Therefore it is hard to reliably compare the baseline situation and early toxicity with the late toxicity we scored. However, the analyses of the subcohort we performed, containing patients who filled out almost all the forms, shows very similar results on almost all analyzed parameters compared to the total cohort. This makes it unlikely that the amount of missing data has a large influence on our outcomes.

## 5 Conclusion

In conclusion, this study shows that treating patients with intermediate- or high-risk localized PCa with RT to 70 Gy in 28 fractions with IMRT/VMAT, using a fiducial-based correction protocol and an ERB leads to good long-term tumor control rates, very acceptable GU- and GI toxicity and a good HRQoL. Furthermore it shows that authors should not only focus on physician-reported toxicity, but also on PROMs and HRQoL, which should be scored in an internationally standardized way. Only then will we be able to truly compare the outcomes of different papers and draw valid conclusions. This is especially important in a time when we are adopting new fractionation schedules based on papers reporting acceptable toxicity profiles. Finally, it shows that using only actuarial incidence rates might cause an overestimation of the toxicity-burden, as prevalence rates show a better correlation with HRQoL-scores.

Funding statement

This research did not receive any specific grant from funding agencies in the public, commercial, or not-for-profit sectors.

Patients’ rights

All procedures followed were in accordance with the ethical standards of the responsible committee on human experimentation (institutional and national) and with the Helsinki Declaration of 1975 (in its most recently amended version). Informed consent was obtained from all patients included in the study.

**Ethical statement**

As the corresponding author, I declare that the work described in the manuscript is unpublished, and is not concurrently being considered for publication elsewhere. I can confirm that the manuscript has been read and approved by all named authors and that there are no other persons who satisfied the criteria for authorship but are not listed. I further confirm that the order of authors listed in the manuscript has been approved by all of us

## Declaration of Competing Interest

The authors declare that they have no known competing financial interests or personal relationships that could have appeared to influence the work reported in this paper.
