# Supplementary material for: Patient reported toxicity and quality of life after hypofractionated high-dose intensity-modulated radiotherapy for intermediate- and high risk prostate cancer
Source: Clin Transl Radiat Oncol. 2021 May 21;29:40–6. doi: 10.1016/j.ctro.2021.05.005 (PMC8170415; doi:10.1016/j.ctro.2021.05.005)
Supplement: Supplementary data 1 [file mmc1.pdf]

Supplementary Figure A.1: prevalence of different grades of toxicity in subscales

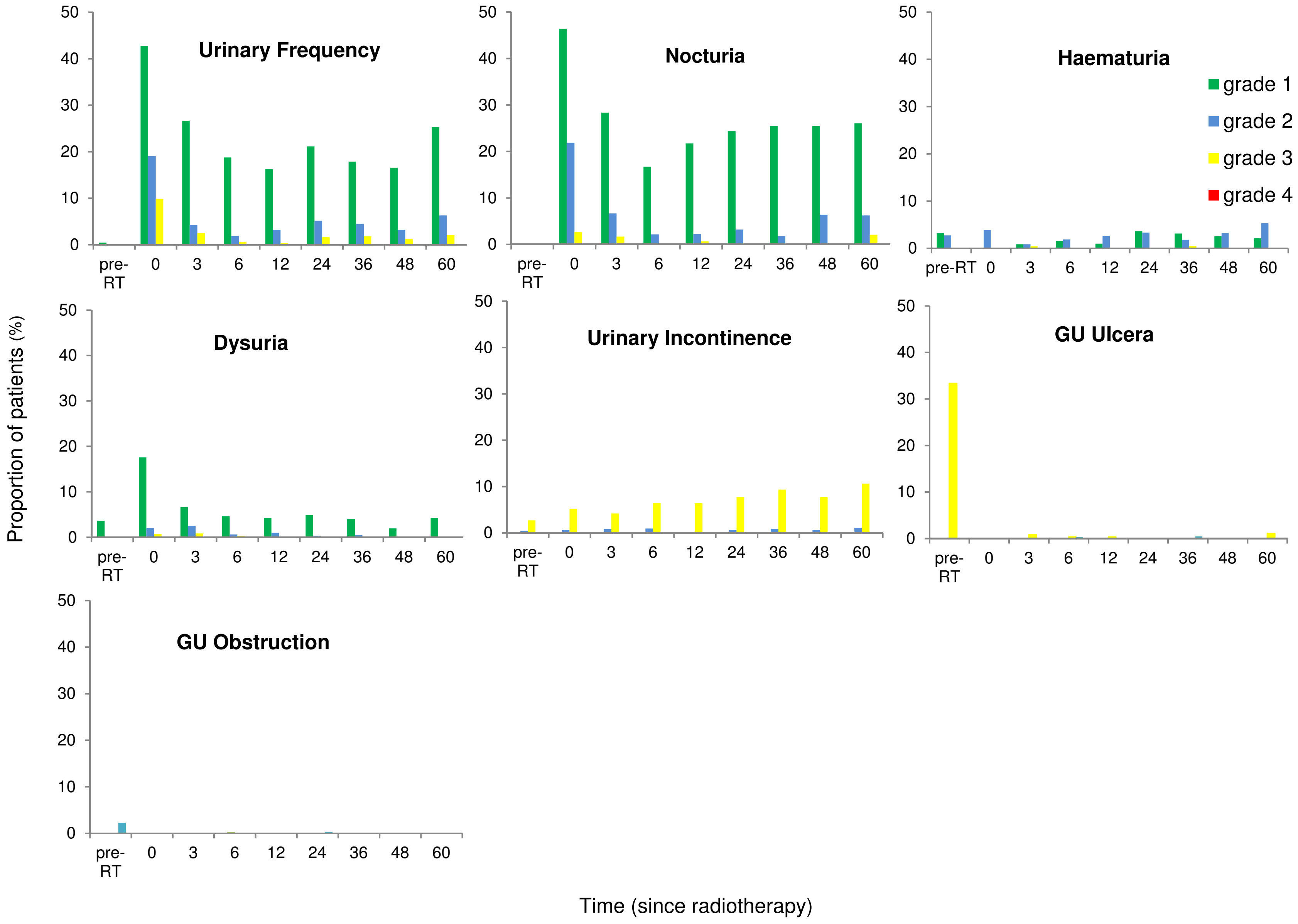

Supplementary Figure A.1: prevalence of different grades of toxicity in subscales

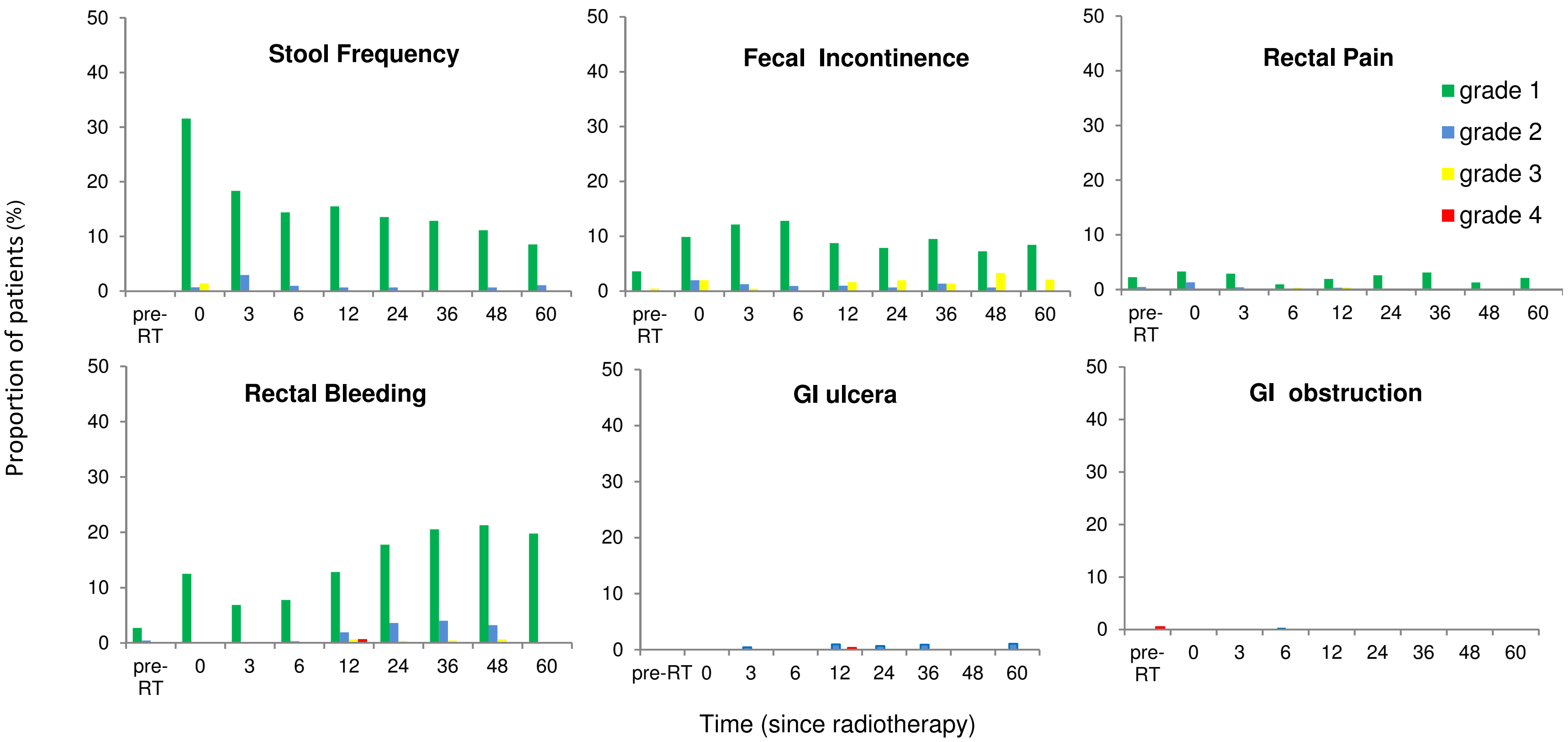

Abbreviations: GU = genitourinary; GI = gastrointestinal
