# Supplementary material for: Patient reported toxicity and quality of life after hypofractionated high-dose intensity-modulated radiotherapy for intermediate- and high risk prostate cancer
Source: Clin Transl Radiat Oncol. 2021 May 21;29:40–6. doi: 10.1016/j.ctro.2021.05.005 (PMC8170415; doi:10.1016/j.ctro.2021.05.005)
Supplement: Supplementary data 3 [file mmc3.pdf]

**Supplementary Table B.1. Mean scores and 95% confidence interval for the EPIC HRQoL questionnaires**

| Bowel Summary | baseline | during RT | 3 months | 6 months | 12 months | 24 months | 36 months | 48 months | 60 months |
|---|---|---|---|---|---|---|---|---|---|
| Mean | 93.3 | 94.7 | 94.2 | 93.8 | 94.1 | 94.9 | 94.7 | 94.5 | 84.6 |
| -95% CI | 95.3 | 85.9 | 90.5 | 92.2 | 91.5 | 91.4 | 91.1 | 91.6 | 91.8 |
| +95% CI | 97.0 | 90.2 | 93.2 | 94.3 | 93.8 | 93.8 | 94.0 | 95.2 | 96.4 |

| Urinary Summary | baseline | during RT | 3 months | 6 months | 12 months | 24 months | 36 months | 48 months | 60 months |
|---|---|---|---|---|---|---|---|---|---|
| Mean | 91.7 | 79.9 | 85.6 | 89.4 | 89.9 | 88.4 | 89.1 | 89.4 | 87.7 |
| -95% CI | 90.4 | 77.7 | 84.0 | 88.0 | 88.6 | 86.9 | 87.5 | 87.3 | 85.1 |
| +95% CI | 93.0 | 82.1 | 87.2 | 90.7 | 91.2 | 89.8 | 90.8 | 91.4 | 90.3 |

---

| Bowel Bother | baseline | during RT | 3 months | 6 months | 12 months | 24 months | 36 months | 48 months | 60 months |
|---|---|---|---|---|---|---|---|---|---|
| Mean | 97.5 | 91.1 | 93.3 | 94.7 | 94.2 | 93.8 | 94.1 | 94.9 | 94.7 |
| -95% CI | 96.6 | 89.2 | 91.9 | 93.6 | 93.0 | 92.5 | 92.7 | 93.1 | 92.3 |
| +95% CI | 98.3 | 93.0 | 94.6 | 95.7 | 95.4 | 95.1 | 95.5 | 96.7 | 97.2 |

| Bowel Function | baseline | during RT | 3 months | 6 months | 12 months | 24 months | 36 months | 48 months | 60 months |
|---|---|---|---|---|---|---|---|---|---|
| Mean | 94.5 | 84.6 | 90.5 | 92.0 | 90.9 | 91.1 | 90.7 | 91.6 | 92.4 |
| -95% CI | 93.4 | 82.1 | 89.1 | 90.9 | 89.6 | 89.9 | 89.2 | 89.8 | 90.2 |
| +95% CI | 95.5 | 87.1 | 91.9 | 93.1 | 92.1 | 92.3 | 92.3 | 93.5 | 94.6 |

---

| Urinary Bother | baseline | during RT | 3 months | 6 months | 12 months | 24 months | 36 months | 48 months | 60 months |
|---|---|---|---|---|---|---|---|---|---|
| Mean | 89.5 | 76.7 | 82.7 | 87.8 | 88.2 | 87.3 | 87.2 | 87.4 | 86.1 |
| -95% CI | 87.9 | 74.4 | 81.0 | 86.4 | 86.8 | 85.7 | 85.4 | 85.1 | 83.1 |
| +95% CI | 91.1 | 79.1 | 84.5 | 89.3 | 89.6 | 88.9 | 89.1 | 89.8 | 89.0 |

| Urinary Function | baseline | during RT | 3 months | 6 months | 12 months | 24 months | 36 months | 48 months | 60 months |
|---|---|---|---|---|---|---|---|---|---|
| Mean | 94.7 | 85.0 | 89.6 | 91.7 | 92.4 | 90.5 | 91.8 | 92.0 | 90.6 |
| -95% CI | 93.5 | 82.5 | 87.7 | 90.3 | 91.1 | 88.9 | 90.2 | 90.2 | 88.0 |
| +95% CI | 96.0 | 87.5 | 91.4 | 93.1 | 93.8 | 92.1 | 93.5 | 93.9 | 93.2 |

| Urinary Incontinence | baseline | during RT | 3 months | 6 months | 12 months | 24 months | 36 months | 48 months | 60 months |
|---|---|---|---|---|---|---|---|---|---|
| Mean | 94.4 | 89.5 | 90.3 | 90.1 | 90.3 | 87.8 | 88.3 | 88.0 | 86.7 |
| -95% CI | 92.8 | 86.2 | 88.3 | 88.3 | 88.5 | 85.8 | 85.8 | 85.4 | 83.0 |
| +95% CI | 96.1 | 92.7 | 92.4 | 91.9 | 92.1 | 89.8 | 90.7 | 90.7 | 90.4 |

| Urinary Irritative | baseline | during RT | 3 months | 6 months | 12 months | 24 months | 36 months | 48 months | 60 months |
|---|---|---|---|---|---|---|---|---|---|
| Mean | 90.6 | 75.2 | 83.5 | 89.5 | 90.1 | 89.6 | 90.1 | 90.6 | 88.6 |
| -95% CI | 89.2 | 72.9 | 81.8 | 88.3 | 88.8 | 88.2 | 88.6 | 88.6 | 86.2 |
| +95% CI | 91.9 | 77.5 | 85.3 | 90.8 | 91.4 | 91.0 | 91.6 | 92.6 | 91.0 |

Abbreviations: EPIC = Expanded Prostate cancer Index Compsite; RT = radiotherapy; -95% CI = lower end of the 95% cofidence interval of the mean; +95% CI = upper end of the 95% confidence interval of the mean
