# Supplementary material for: Patient reported toxicity and quality of life after hypofractionated high-dose intensity-modulated radiotherapy for intermediate- and high risk prostate cancer
Source: Clin Transl Radiat Oncol. 2021 May 21;29:40–6. doi: 10.1016/j.ctro.2021.05.005 (PMC8170415; doi:10.1016/j.ctro.2021.05.005)
Supplement: Supplementary data 4 [file mmc4.pdf]

Supplementary table B.2. Uni- and multivariate analyses of the prognostic value for predicting long-term grade ≥2 toxicity

	Univariate		Multivariate	
	HR (95% CI)	p value	HR (95% CI)	p value
**GU toxicity**				
Age (≥ 70 year)	1.22 (0.83-1.79)	0.307		
Gleason Score (≥7)	1.38 (0.89-2.14)	0.155		
Risk-group (high-risk)	0.86 (0.59-1.28)	0.463		
Treatment group (including (base of) SVs)	2.05 (1.12-3.74)	**0.017**	2.77 (0.998-7.68)	0.051
Pre-treatment PSA (≥20 ng/mL)	0.88 (0.54-1.42)	0.593		
Neoadjuvant hormonal therapy (>6 months)	1.45 (1.00-2.11)	0.796		
Oral anticoagulants	1.47 (0.85-2.54)	0.169		
Pelvic surgery	1.89 (1.30-2.76)	**0.001**	1.92 (1.14-3.24)	**0.014**
Baseline symptoms of GU toxicity (≥ grade 1)	3.43 (1.90-6.19)	**<0.001**	3.16 (1.75-5.73)	**<0.001**
**GI toxicity**				
Age (≥ 70 year)	1.42 (0.81-2.51)	0.226		
Gleason Score (≥7)	2.05 (1.00-4.20)	**0.050**	4.04 (1.22 - 13.36)	**0.022**
Risk-group (high-risk)	1.49 (0.80-2.79)	0.212		
Treatment group (including (base of) SVs)	2.12 (0.84-5.34)	0.110		
Pre-treatment PSA (≥20 ng/mL)	1.49 (0.81-2.75)	0.199		
Neoadjuvant hormonal therapy (>6 months)	1.38 (0.61-3.13)	0.436		
Oral anticoagulants	1.46 (0.66-3.24)	0.354		
Pelvic surgery	1.32 (0.77-2.27)	0.310		
Baseline symptoms of GU toxicity (≥ grade 1)	2.87 (1.18-7.02)	**0.021**	3.34 (1.36-8.21)	**0.009**

Abbreviations: GU = genitourinary; GI = gastrointestinal; SV = seminal vesicles; HR = hazard ratio; CI = confidence interval
