# Supplementary material for: Patient reported toxicity and quality of life after hypofractionated high-dose intensity-modulated radiotherapy for intermediate- and high risk prostate cancer
Source: Clin Transl Radiat Oncol. 2021 May 21;29:40–6. doi: 10.1016/j.ctro.2021.05.005 (PMC8170415; doi:10.1016/j.ctro.2021.05.005)
Supplement: Supplementary data 5 [file mmc5.pdf]

Supplementary tables B.3. Comparison of outcomes between the subcohort and the total cohort

FFF-rates (%):

	36 months	60 months
total cohort	92.0	83.5
subcohort	96.8	88.2

Prevalence rates (%):

	baseline	6 months	12 months	24 months	36 months	48 months	60 months
GU grade ≥2 toxicity total cohort	7	12.1	12.5	15.8	16.3	16.8	22.1
GU grade ≥2 subcohort	1.7	9.8	9.4	10.5	14.7	15.4	23.9
GI grade ≥2 toxicity total cohort	1.3	2.2	6.1	7.1	6.3	7.8	3.2
GU grade ≥2 toxicity subcohort	1.8	3.2	6.4	5.4	7.5	9.2	3.1

Cumulative incidence rates (%):

	36 months	60 months
GU grade ≥2 toxicity total cohort	26.3	43.5
GU grade ≥2 subcohort	23.4	40.2
GI grade ≥2 toxicity total cohort	15.0	18.5
GU grade ≥2 toxicity subcohort	12.9	15.6

EPIC HRQoL scores:

	baseline	6 months	12 months	24 months	36 months	48 months	60 months
urinary summary total cohort	91.7	89.4	89.9	88.4	89.1	89.4	87.7
urinary summary subcohort	93.3	89.5	89.3	89.3	88.8	88.9	86.9
bowel summary total cohort	96.1	93.3	92.7	92.6	92.5	93.4	94.1
bowel summary subcohort	95.8	93.9	93.9	94.5	92.9	93.3	94.2
