# Supplementary material for: Patient reported toxicity and quality of life after hypofractionated high-dose intensity-modulated radiotherapy for intermediate- and high risk prostate cancer
Source: Clin Transl Radiat Oncol. 2021 May 21;29:40–6. doi: 10.1016/j.ctro.2021.05.005 (PMC8170415; doi:10.1016/j.ctro.2021.05.005)
Supplement: Supplementary data 6 [file mmc6.pdf]

The questions in this document consist of the Expanded Prostate cancer Index Composite (EPIC) combined with complementary questions. These complementary questions were translated from Dutch to English for the purpose of this publication.

URINARY FUNCTION
This section is about your urinary habits. Please consider ONLY THE LAST 4 WEEKS

1x. Over the **past 4 weeks,** how often did you urinate <u>during the day</u>, compared to the situation before the radiation therapy?

About as often as before the radiation	1
Twice as often as before the radiation	2
More than twice as often as before the radiation	3
A lot more, over 12 times during the day	4

How many times do you urinate during the day? …….. times a day

1y. Over the **past 4 weeks,** how often did you urinate <u>during the night</u>, compared to the situation before the radiation therapy?

About as often as before the radiation	1
Twice as often as before the radiation	2
More than twice as often as before the radiation	3
Over 8 times during the night	4

How many times do you urinate during the day? …….. times a night

1. Over the **past 4 weeks**, how often have you leaked urine?

More than once a day	1
About once a day	2
More than once a week	3
About once a week	4
Rarely or never	5

2. Over the **past 4 weeks**, how often have you urinated blood?

More than once a day	1
About once a day	2
More than once a week	3
About once a week	4
Rarely or never	5

2b. How severe was the bloodless in the urine over **the past 4 weeks**?

No blood loss	1
No visible blood loss, but positive test for blood loss in the urine	2
Sometimes blood loss OR received treatment for blood loss In het urine (other than a transfusion)	3
Needed a blood transfusion	4

3. Over the **past 4 weeks**, how often have you had pain or burning with urination?

More than once a day	1
About once a day	2
More than once a week	3
About once a week	4
Rarely or never	5

3b. How bad was the pain or the burning sensation with urination **the past 4 weeks**?

(Almost) No pain 1
Slightly painful, for which I sometimes used pain killers 2
Moderate pain, for which I used pain killers more than twice a week or used (local) anaesthetics (like lidocaine gel) 3
Severe pain, for which I used (local) anaesthetics more than twice a week 4

3c. Did you, over the **past 4 weeks,** have any trouble with urinating of are you treated for this?

No trouble or no treatment 1
Yes, but not treated or only treated with medication* 2
Yes, treated with mechanical dilation for 1 or 2 times 3
Yes, treated with more than 2 mechanical dilations 4
Yes, underwent surgery for this problem 5
* name of the medication: _______________

3d. Did you, **__before__ the past 4 weeks,** have any trouble with urinating or are you treated for this?

No, no trouble or no treatment 1
Yes, Yes, but not treated or only treated with medication* 2
Yes, treated with (mechanical) dilation for 1 or 2 times 3
Yes, treated with more than 2 (mechanical) dilations 4
Yes, underwent surgery for this problem 5

3e. If you were treated for having trouble urinating: what was the date of this treatment: .... - …. - …….

4. Which of the following best describes your urinary control **during the last 4 weeks**?

No urinary control whatsoever 1
Frequent dribbling 2
Occasional dribbling 3
Total control 4

4b. How often did you use diapers or pads to intercept unwanted loss of urine **past 4 weeks**?

Never 1
2 or less days a week 2
3 days or more a week, but not every day 3
Every day 4

5. How many pads or adult diapers per day did you usually use to control leakage **during the last 4 weeks**?

None 0
1 pad a day 1
2 pads a day 2
3 or more pads a day 3

5b. A possible complication of radiation is ulcers in the urinary bladder. Are you, in the **past 4 weeks**, treated for these ulcers?

No 1
Yes, with oxygen treatment in the urinary bladder 2
Yes, with surgery 3

5c. Were you, **__before__ the past 4 weeks**, ever treated for these ulcers?

No 1
Yes, with medication 2
Yes, with oxygen treatment in the urinary bladder 3
Yes, with surgery

5d. If you were ever treated for these ulcers, what was the date of this treatment: …. - …. - ……..

6. How big a problem, if any, has each of the following been for you **during the last 4 weeks**?

(Circle one number on each line)

	No Problem	Very Small Problem	Small Problem	Moderate Problem	Big Problem
a. Dripping or leaking urine	0	1	2	3	4
b. Pain or burning on urination	0	1	2	3	4
c. Bleeding with urination	0	1	2	3	4
d. Weak urine stream or incomplete emptying	0	1	2	3	4
e. Waking up to urinate	0	1	2	3	4
f. Need to urinate frequently during the day	0	1	2	3	4

7. Overall, how big a problem has your urinary function been for you **during the last 4 weeks**?

No problem 1
Very small problem 2
Small problem 3
Moderate problem 4
Big problem 5

BOWEL HABITS
The next section is bout your bowel habits and abdominal pain.
Please consider ONLY THE LAST 4 WEEKS.

8. How often have you had rectal urgency (felt like I had to pass stool, but did not) **during the last 4 weeks**?

More than once a day 1
About once a day 2
More than once a week 3
About once a week 4
Rarely or never 5

9. How often have you had uncontrolled leakage of stool or faeces during the **past 4 weeks**?

More than once a day 1
About once a day 2
More than once a week 3
About once a week 4
Rarely or never 5

9b. How bad was, **over the last 4 weeks**, the uncontrolled leakage of stool or faeces?

No uncontrolled leakage 1
Mild, no pads or other materials required 2
Use of pads or other materials required for several, but not daily 3
Daily use of pads or other materials 4

10. How often have you had stools (bowel movements) that were loose or liquid (no form, watery, mushy) **during the last 4 weeks**?

Never 1
Rarely 2
About half the time 3
Usually 4
Always 5

10b. Did you, **over the last 4 weeks**, have any obstipation caused by tightening of the bowels (not by thickening of the stools/faeces)

No	1
Yes, but I did not have any treatment for these problems	2
Yes, I had (mechanical) dilation 1-2 times	3
Yes, I had (mechanical) dilation more than 2 times	4
Yes, I had surgery for this problem	5

10c. Did you**, <u>before</u> the past 4 weeks**, experience obstipation caused by tightening of the bowels (not by thickening of the stools/faeces)

No	1
Yes, but I did not have any treatment for these problems	2
Yes, I had (mechanical) dilation 1-2 times	3
Yes, I had (mechanical) dilation more than 2 times	4
Yes, I had surgery for this problem	5

10d. If you were treated, on which date was this treatment: …. - …. - ……..

11. How often have you had bloody stools **during the last 4 weeks**?

Never	1
Rarely	2
About half the time	3
Usually	4
Always	5

11b. How bad were these bloody stools during the **past 4 weeks**?

Did not occur	1
Mild, no treatment	2
Moderate, I underwent treatment (no blood transfusion!)	3
Severe, I underwent blood transfusion OR more than 2 treatments with oxygen or otherwise	4
Very severe, I have been hospitalized for this	5

11c. If you underwent treatment for bloody stools, what was the date of this treatment: …. - …. - ……

12. How often have your bowel movements been painful **during the last 4 weeks**?

Never	1
Rarely	2
About half the time	3
Usually	4
Always	5

12b. How severe was this pain during bowel movements in the **last 4 weeks?**

(almost) no pain	1
Mild pain, for which I sometimes used painkillers	2
Moderate pain, for which I used painkillers more than 2 times a week OR used (local) anaesthetics	3
Severe pain, for which I used (local) anaesthetics more than two times a week	4

13. How many bowel movements have you had on a typical day **during the last 4 weeks**?

Two or less	1
Three to four	2
Five or more	3

13b. Is this more frequent than before the radiation and did you, **in** the last 4 weeks, use anti-diarrheal medication for
this problem?

Not more frequent than before the radiation	1
About twice as frequent, did not use medication	2
Many times more frequent, I used a maximum of 2 anti-diarrheal medications a day	3
Many times more frequent, I used more than 2 anti-diarrheal medications a day	4

13c. Did you, **in the past 4 weeks**, worry about your bowel movements?

Yes, very much	1
Yes, quite often	2
Yes, but just a little	3
No, I did not worry	4

14. How often have you had crampy pain in your abdomen, pelvis or rectum **during the last 4 weeks**?

More than once a day	1
About once a day	2
More than once a week	3
About once a week	4
Rarely or never	5

14b. A possible complication of radiation is an inflammation or ulcer of the bowel. Are you, **in the last 4 weeks**, treated for one of these problems?

No	1
Yes, with oral medication (steroids)	2
Yes, with steroids injected in the bowel or with oxygen treatments	3
Yes, I underwent surgery for this problem	4

14c. Are you, **<u>before</u> the last 4 weeks**, ever treated for these problems (inflammation/ulcers of the bowel)
weeks, treated for one of these problems?

No	1
Yes, with oral medication (steroids)	2
Yes, with steroids injected in the bowel or with oxygen treatments	3
Yes, I underwent surgery for this problem	4

14d. If you were treated for this problem, what was the date of this treatment: …. - …. - ……..

15. How big a problem, if any, has each of the following been for you? (Circle one number on each line)

	No Problem	Very Small Problem	Small Problem	Moderate Problem	Big Problem
a. Urgency to have bowel movement	0	1	2	3	4
b. Increased frequency of bowel movements	0	1	2	3	4
c. Watery bowel movements	0	1	2	3	4
d. Losing control of your stools	0	1	2	3	4
e. Bloody stools	0	1	2	3	4
f. Abdominal/ Pelvic/Rectal pain	0	1	2	3	4

16. Overall, how big a problem have your bowel habits been for you **during the last 4 weeks**?

No problem	1
Very small problem	2
Small problem	3
Moderate problem	4
Big problem	5
